# Arthroscopic Assisted Acromioclavicular Joint Stabilization Using a Tensionable Suture Button Construct and Cerclage Tape Augmentation

**DOI:** 10.1002/atn2.70036

**Published:** 2026-06-25

**Authors:** Sean Wei Loong Ho, Yu Qian Cheong, T. Jegathesan, Lester Teong Jin Tan, Derrick Junhong Guo, Keng Thiam Lee, Marcus Josef Lee

**Affiliations:** ^1^ Department of Orthopaedic Surgery Tan Tock Seng Hospital Singapore Singapore; ^2^ Yong Loo Lin School of Medicine National University of Singapore Singapore Singapore; ^3^ Department of Orthopaedic Surgery Woodlands Hospital Singapore Singapore

## Abstract

Surgical stabilization of the acromioclavicular joint can be achieved using a variety of techniques. Coracoclavicular fixation with tapes, sutures, and button systems is one of the surgical methods used to restore stability of the acromioclavicular joint. There is currently no definitive superior technique for acromioclavicular joint fixation, and complications such as loss of reduction and implant‐related issues remain a concern. The purpose of this Technical Note is to describe a reproducible surgical technique for acromioclavicular joint stabilization that employs a tensionable coracoclavicular button system augmented with cerclage suture tapes. This construct allows for precise tensioning, improved stability, and the potential to minimize the need for additional fixation or hardware removal.

## Graphical abstract

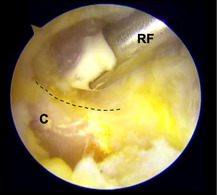

**VIDEO 1:** The patient is positioned in a beach chair position and the arm is draped free. A standard posterior viewing portal is used. A spinal needle is placed parallel to the subscapularis for creation of the anterolateral portal. Initial exposure of the coracoid body is performed by viewing from the posterior portal and working from the AL portal with the radiofrequency (RF) wand (Smith & Nephew, Andover, MA, USA). Visualisation can be improved by viewing from the AL portal. The exposure of the rest of the coracoid base can be performed working with the RF from the anterior portal. Once the coracoid is adequately exposed, a longitudinal incision is made over the distal clavicle. The authors prefer an open exposure to allow for improved reduction. The medial and lateral aspect of the coracoid is determined with a spinal needle. Independent drilling of the coracoid is performed using an anterior cruciate ligament tibial jig (Smith & Nephew, Andover, MA, USA) introduced from the anterior portal. The anterior cruciate ligament jig is set at 60° and the bullet trocar is positioned centrally on the top of the coracoid with the tip aimer at the base of the coracoid. It is important to recheck the medial and lateral aspect of the coracoid prior to drilling to ensure central drilling and avoid iatrogenic fracture. Once drilled through, a looped wire is passed through the drill hole. Retrograde shuttling of the glenoid bone loss button system is performed from the anterior portal. It is imperative to ensure that the button peg sits firmly into the drill hole at the base of the coracoid. A switching stick is then inserted medial to the coracoid. This passage is enlarged with a cannula dilator. A looped suture is passed through the dilator and one end of 2 tapes (Smith & Nephew, Andover, MA, USA) is passed medially. This same procedure is repeated for the lateral aspect to ensure that 2 tapes pass under the coracoid. If a graft is desired, it can also be passed in this same fashion. Once the button system, tapes and graft are passed under the coracoid, attention is turned to the distal clavicle. Based on X‐ray measurements of the unaffected side, 2 mm drill holes are drilled at the medial and lateral aspect of the coracoid, with an additional drill hole in the middle. The GBL button system is passed into the centre hole whilst each end of the tapes are passed into the medial and lateral holes. One end of the graft can be passed under the clavicle. A tensioner is then used to cinch down the button on the top of the clavicle at 80 N. The knot is then tied to secure the tension. The ends of the tapes are subsequently tied. The graft is secured in a standard figure‐of‐8 configuration with sutures. Finally, 2 mm drill holes are drilled from an anterior to posterior direction on the distal clavicle and acromion. A single tape is passed through them in a figure‐of‐8 configuration, and the ends of the tape are tied on a button anterior to the distal clavicle. The wound is then closed in layers, taking care to perform adequate fascial closure. 1‐year postoperative X‐ray shows stable reduction with no expansion of bony tunnels. Video content can be viewed at https://doi.org/10.1002/atn2.70036.

Acromioclavicular joint injuries are typically caused by direct trauma to the superior aspect of the shoulder when the arm is adducted, or by indirect trauma resulting from a humeral head shift impacting the acromion.^1^, ^2^ In athletes participating in contact sports, acromioclavicular joint injuries can constitute up to 50% of all shoulder injuries.^2^ The Rockwood classification is a well‐established system that grades the severity of displacement of the distal clavicle from the acromion, and thus indicating the extent of associated ligamentous injury; and also guides the management of acromioclavicular joint injuries.^3^ Typically, Rockwood types I and II are managed conservatively, types IV to VI are treated surgically, whereas the treatment of type III injuries remains controversial.^4^

Although numerous surgical techniques have been described for high‐grade acromioclavicular joint separations, no consensus exists regarding a gold standard of treatment.^5^ Implants such as Kirschner wires (K‐wires), Bosworth screws, or hook plates can achieve adequate reduction. However, they are associated with complications such as implant prominence, failure and migration, shoulder impingement and acromial erosion, often necessitating subsequent removal.^6^, ^7^ In addition, a loss of reduction remains a concern.^8^, ^9^

Coracoclavicular fixation using a suspensory device has been gaining popularity. The use of a suture button or suture cerclage is now preferred, as it eliminates the need for secondary implant removal, and suture buttons have been reported to be biomechanically comparable to native ligaments.^10^, ^11^ Complementing suspensory fixations with a tensioning device allows the surgeon to precisely control reduction and achieve stable acromioclavicular joint fixation without the need for temporary fixation or additional assistance.^12^

The aim of this Technical Note is to describe a surgical technique for acromioclavicular joint stabilization using a combination of a tensionable suture button system with cerclage sutures.

## SURGICAL TECHNIQUE

### Preoperative Patient Positioning

The surgical procedure is performed with the patient under general anesthesia (Video 1). The authors’ preference is to perform the surgery with the patient in a beach chair position. The affected shoulder is cleaned and the upper limb is draped in a sterile stockinette. A mechanical arm‐holder can be used if desired. Image intensifier is placed on the operated side of the patient.

### Arthroscopic Assessment and Preparation

**FIGURE 1:**
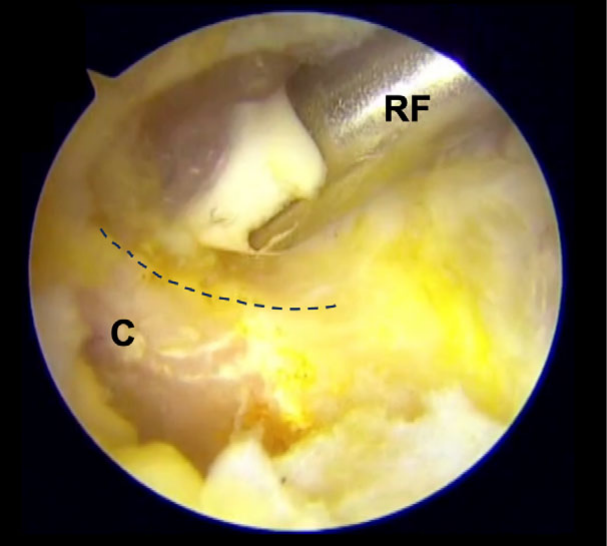
Right Shoulder. Beach chair position. Viewing from posterior portal: The coracoid (C) is visualised and the radiofrequency (RF) ablator is inserted via the anterior portal to clear the margins of the coracoid. The lateral margin of the coracoid is delineated here (dashed line).

**FIGURE 2:**
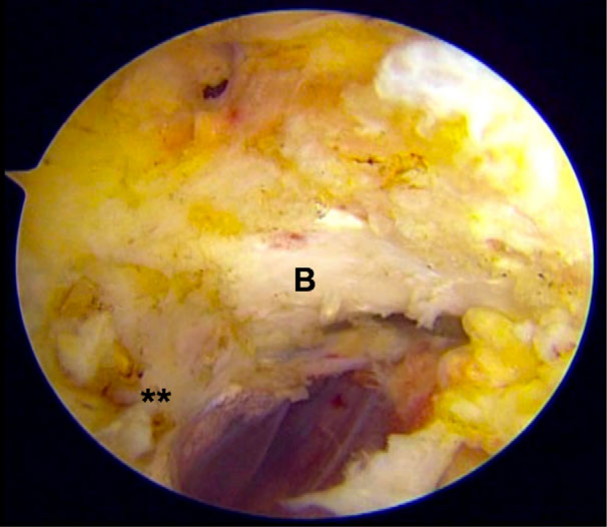
Right shoulder. Beach chair position. Viewing from anterolateral (AL) portal: Additional visualisation of the coracoid (C) can be achieved by viewing from the AL portal and working from the anterior portal. After clearance of the soft tissue, the base (**) and body (B) of the coracoid is clearly visible.

This procedure uses a standard posterior portal, anterior portal and antero‐lateral (AL) portal. The posterior portal is created and a 30° scope is used for viewing. A diagnostic arthroscopy is performed to ensure that there are no concomitant pathologies. An AL portal is created and the radiofrequency ablator is used to expose the medial and lateral edges of the coracoid body. (Figure 1). An anterior portal is made lateral to the conjoint tendon. The scope is then switched to the AL portal for improved visualization of the coracoid base. (Figure 2). The RF can be used from the anterior portal to clear soft tissue from the base of the coracoid.

### Exposure of the Acromioclavicular Joint

**FIGURE 3:**
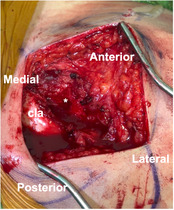
Right shoulder. Beach chair position. An open incision is made centered over the distal clavicle (cla). The open incision allows for better visualisation and reduction of the acromioclavicular joint. The anterior deltoid is released so that the superior, medial and lateral margins of the coracoid (*) may be identified.

A horizontal incision is made over the distal clavicle and acromioclavicular joint. (Figure 3). The anterior deltoid is dissected off the anterior clavicle so that the superior aspect of the coracoid can be palpated. An open approach is preferred by the authors so that scar tissue from the acromioclavicular joint can be excised to aid in effective reduction.

### Insertion of the Coracoclavicular Button System

**FIGURE 4:**
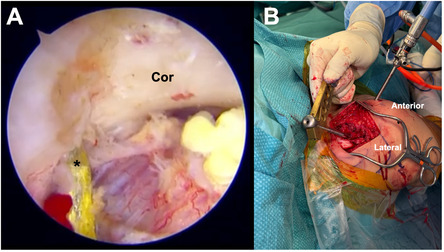
Right shoulder. Beach chair position. (A) Viewing from anterolateral (AL) portal: An anterior cruciate ligament (ACL) jig (Smith & Nephew, Andover, MA, USA) set at 60° is inserted via the anterior portal, with the tip aimer (*) placed at the base of the coracoid. [Cor ‐ coracoid]. (B) External view: The trocar for the jig sits directly centered over the superior aspect of the coracoid. Medial and lateral margins of the coracoid are identified to prevent iatrogenic fracture of the coracoid.

**FIGURE 5:**
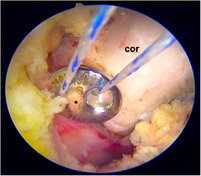
Right shoulder. Beach chair position. Viewing from anterolateral (AL) portal: The Glenoid Bone Loss system (Glenoid Bone Loss system, Smith & Nephew, Andover, MA, USA) is passed through the anterior portal and shuttled into the drill hole of the coracoid (cor). It is important to ensure that the pegged button (*) sits within the drill hole at the base of the coracoid.

Viewing from the AL portal, an anterior cruciate ligament jig (Smith & Nephew, Andover, MA, USA) set at 60° is inserted via the anterior portal. (Figure 4). The tip of the jig is placed in the mid‐section of the base of the coracoid and the bullet trocar is set against the top of the coracoid (Table 1). Care is taken to position the tip of the jig in the centre of the coracoid to avoid iatrogenic fracture. A 2 mm drill hole is made under direct arthroscopic visualization. A rigid passing wire is placed through this hole from top to bottom, and the button system (Glenoid Bone Loss system, Smith & Nephew, Andover, MA, USA) is shuttled through the coracoid, with the button deploying at the base of the coracoid (Figure 5).

**TABLE 1:** Pearls and Pitfalls

Pearls	‐ A tip aiming anterior cruciate ligament (ACL) jig (Smith & Nephew, Andover, MA, USA) set at 60° (or more) can be used to safely drill the coracoid tunnel under direct arthroscopic vision
	‐ Preoperative measurement of the medial and lateral coracoid on the un‐injured side can determine the ideal position of the tunnels to be drilled for cerclage suture
	‐ Rigid wire passer or looped wire passer (Smith & Nephew, Andover, MA, USA) can be used to easily pass the sutures into the clavicle
	‐ After reduction with the tensioner, intra‐operative imaging should be performed to assess for adequacy of reduction or over‐reduction
Pitfalls	‐ Ensure that the ACL jig is sitting in the center of the coracoid before drilling to avoid iatrogenic fracture
	‐ Avoid over‐tensioning the suture button construct to prevent the complications of over‐reduction or suture breakage

### Passing of Cerclage Suture Tapes

**FIGURE 6:**
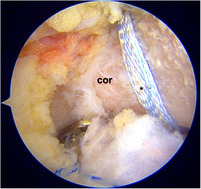
Right shoulder. Beach chair position. Viewing from anterolateral (AL) portal: The ends of two suture tapes (*) are passed medial and lateral to the coracoid (cor) to ensure that the tape is looped around the coracoid. If a graft is desired in chronic cases, the graft can be passed in the same fashion as the suture tapes.

Viewing from the AL portal, a switching stick is used to determine the medial aspect of the coracoid. Once its position is confirmed arthroscopically, a dilator is placed over the switching stick, and a rigid passing wire is passed through it. This is retrieved using a suture retriever from the anterior portal. Two suture tapes (Smith & Nephew, Andover, MA, USA) are shuttled medial to the coracoid. This same procedure is performed lateral to the coracoid, and the other end of the 2 suture tapes are shuttled laterally (Figure 6). These suture tapes will be used for a cerclage fixation. If a graft is desired in chronic cases, the graft can be passed in the same fashion as the suture tapes.

### Passing of Button System and Sutures Into the Clavicle

**FIGURE 7:**
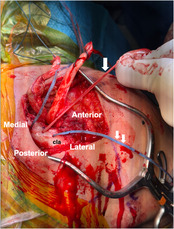
Right shoulder. Beach chair position. Three 2 mm tunnels are drilled into the clavicle. The glenoid bone loss sutures (single white arrow) are placed within the middle 2 mm drill hole, whilst each end of the suture tape (double white arrow) is placed into the medial and lateral 2 mm drill holes. (cla, clavicle.)

Using measurements from the plain radiograph of the unaffected side, three 2 mm holes are drilled into the clavicle. The positions of which are medial to the coracoid, centred over the coracoid and lateral to the coracoid—approximately 4.5 cm from ACJ, 4 cm from ACJ, and 3 cm from ACJ. The suture tapes are passed into the medial and lateral drill holes to form a cerclage. The button system sutures are passed into the central 2 mm drill hole (Figure 7). Once it is passed through, a loose button is placed over the tensionable button system sutures.

### Reduction and Tensioning

**FIGURE 8:**
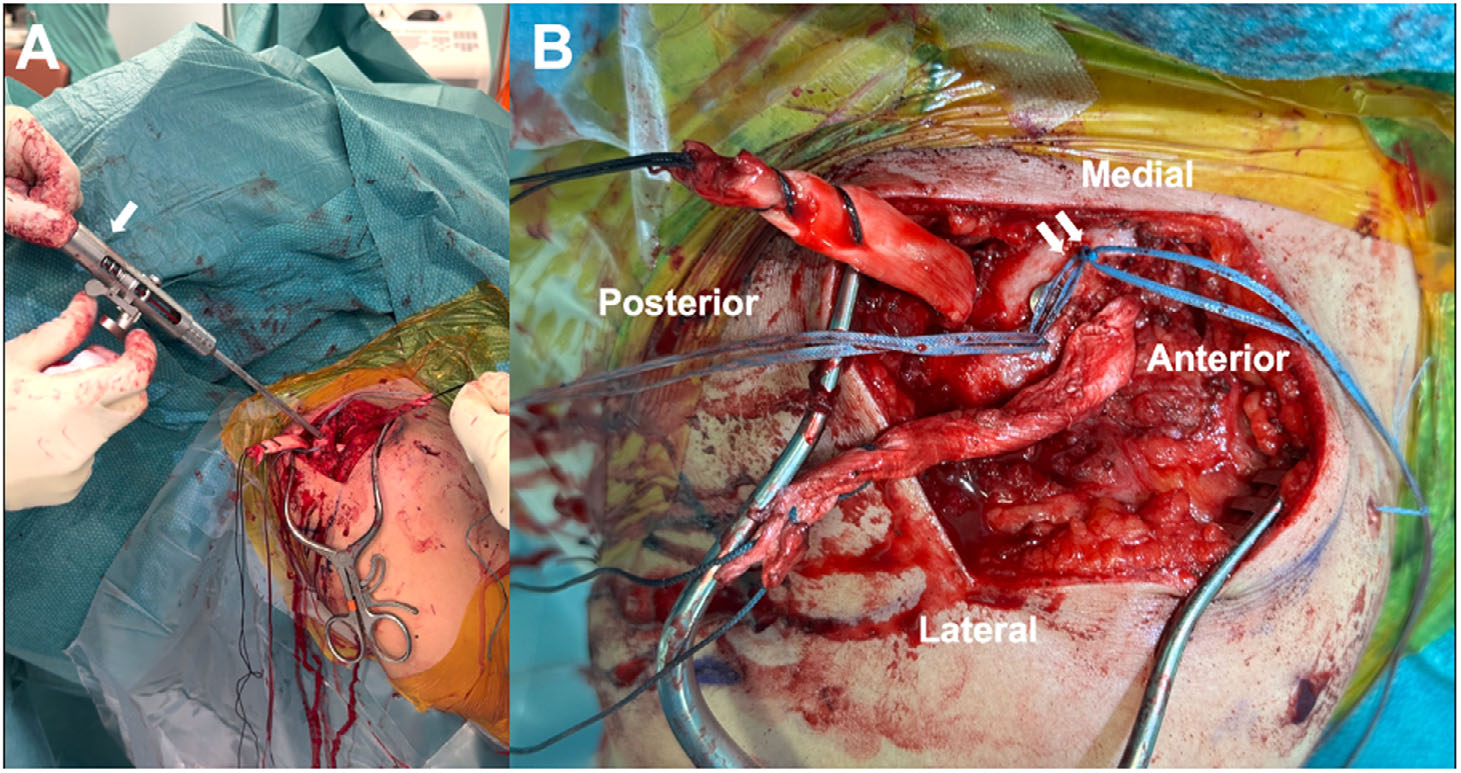
Right shoulder. Beach chair position. (A) The tensioner (white arrow) is used to reduce the clavicle to its appropriate position. (B) The *Nice* knot is then secured and the cerclage suture tapes are then tied for reinforcement of the construct (double white arrow).

A *Nice* knot is tied using the button system sutures and the tensioner is used for reduction of the clavicle. A force of 80 N is used. Thereafter, an image intensifier is used to confirm adequate reduction and the *Nice* knot is secured. The cerclage suture tapes are then tied for reinforcement of the construct (Figure 8).

### Acromioclavicular Joint Stabilization

**FIGURE 9:**
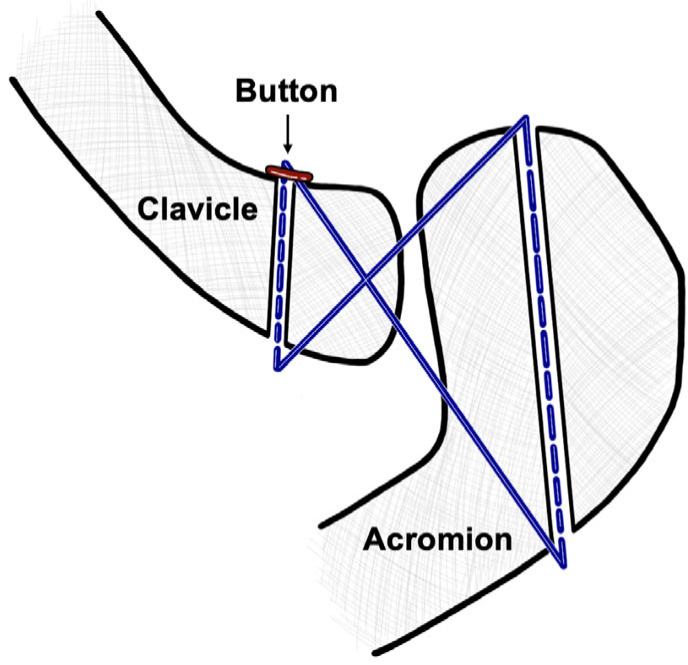
Illustration. Two horizontal 2 mm tunnels are made in the distal clavicle and across the acromion. For horizontal stability, one suture tape (blue line) (Smith & Nephew, Andover, MA, USA) is passed through the distal clavicle and acromion in a figure‐of‐8 configuration. This is tied over a button on the anterior surface of the distal clavicle to reduce the risk of cut‐through of the bone by the tapes.

Two horizontal 2 mm tunnels are made in the distal clavicle and across the acromion. One suture tape is passed through it in a figure‐of‐8 configuration and this is tied over a button on the anterior surface of the distal clavicle to reduce the risk of cut‐through of the bone by the tapes (Figure 9).

### Postoperative Protocol

The operated upper extremity is placed in an arm sling. Strict arm sling usage is observed for 6 weeks. Thereafter, range‐of‐motion exercises and full weight bearing is allowed. A progressive strengthening program is then implemented.

## DISCUSSION

Suture fixation for acromioclavicular joint separation is a well‐established surgical technique, offering the advantages of avoiding implant removal and reducing hardware‐related complications. In addition, fixation with suture buttons has been shown to provide high biomechanical stability.^13^

Arthroscopic‐assisted techniques confer specific benefits. Although coracoid fixation may be undertaken via an open approach, the use of arthroscopy enables more precise button placement and safer passage of suture tapes, thereby minimizing the risk of neurological injury.^14^

In our method, small‐diameter tunnels (2 mm) are created in the clavicle. This small diameter reduces the risk of iatrogenic fracture and does not significantly alter the clavicle's load‐to‐failure compared with its intact state.^15^ Additionally, separate tunnels are drilled in the clavicle and coracoid, eliminating the need for a “reduction‐first” approach. This noncollinear drilling technique not only reduces technical difficulty, since using a jig to drill directly from the clavicle to the coracoid can be challenging, but also minimizes the risk of iatrogenic fractures of both the clavicle and coracoid.^16^

Our method of cerclage suture tape augmentation, using tunnels drilled in the clavicle on either side of the coracoid, aims to replicate the native positions of the coracoclavicular ligaments. However, a biomechanical study showed that acromioclavicular stabilization using coracoid loop devices alone can anteriorly displace the clavicle.^17^ This limitation is addressed in our technique, as suture buttons passed directly through tunnels in both the clavicle and coracoid restore stability in the anterior, posterior, superior, and inferior directions to values comparable with the native joint^17^ (Table 2).

**TABLE 2:** Advantages and Disadvantages of This Technique

Advantages	‐ This arthroscopic‐assisted technique ensures that the coracoid tunnel is centred to reduce the risk of iatrogenic fracture. It also ensures that the suture tapes are appropriately placed under the coracoid
	‐ This technique requires only small diameter tunnels (2 mm) which reduces the risk of iatrogenic fracture of the clavicle
	‐ This tensioning system allows for a single surgeon to perform the reduction without the need for temporary Kirschner wire fixation
Disadvantages	‐ This is an off‐label use of suture button tensioning system
	‐ Suture‐button devices may predispose clavicular tunnel widening

This tensionable system, combining both suture button and suture cerclages, provides horizontal and vertical stability of the acromioclavicular joint. The technique is straightforward and reproducible, allowing for adequate and sustained reduction, and can be performed by a single surgeon without assistance.

## DISCLOSURES

The author (S.W.L.H.) declares the following financial interests/personal relationships which may be considered as potential competing interests: S.W.L.H. reports a relationship with Smith & Nephew that includes: speaking and lecture fees; reports a relationship with DePuy Synthes Mitek Sports Medicine that includes: speaking and lecture fees. The other authors (Y.Q.C., J.T., L.T.J.T., D.J.G., K.T.L., M.J.L.) declare that they have no known competing financial interests or personal relationships that could have appeared to influence the work reported in this paper.

## References

[1] Frank RM , Cotter EJ , Leroux TS , Romeo AA . Acromioclavicular joint injuries. J Am Acad Orthop Surg. 2019;27:e775‐e788. 31008872 10.5435/JAAOS-D-17-00105

[2] Adra M , Mohamed Haroon A , Milchem H , et al. Operative versus nonoperative management of high‐grade acromioclavicular injuries: A systematic review and meta‐analysis. Cureus. 2024;16:e76682. 39898132 10.7759/cureus.76682 PMC11785354

[3] Rockwood CA Jr . Fractures and dislocations of the shoulder. eds. Rockwood CA Jr , Green DP , Fractures in Adults. Philadelphia, PA: Lippincott, 1984;860‐910.

[4] Tamaoki MJ , Belloti JC , Lenza M , Matsumoto MH , Gomes dos Santos JB , Faloppa F . Surgical versus conservative interventions for treating acromioclavicular dislocation of the shoulder in adults. Cochrane Database Syst Rev. 2010;2010:CD007429. 20687087 10.1002/14651858.CD007429.pub2 PMC6465032

[5] Ardebol J , Hwang S , Horinek JL , Parsons BO , Denard PJ . Arthroscopically assisted tensionable cerclage reconstruction of an acromioclavicular separation with combined fixation of the coracoclavicular and acromioclavicular ligaments. Arthrosc Tech. 2023;12:e321‐e327. 37013023 10.1016/j.eats.2022.11.010 PMC10066044

[6] Lin HY , Wong PK , Ho WP , Chuang TY , Liao YS , Wong CC . Clavicular hook plate may induce subacromial shoulder impingement and rotator cuff lesion ‐ dynamic sonographic evaluation. J Orthop Surg Res. 2014;9:6. 24502688 10.1186/1749-799X-9-6 PMC3922330

[7] Tiefenboeck TM , Popp D , Boesmueller S , Joestl J , Orthner E , Hajdu S . Acromioclavicular joint dislocation treated with Bosworth screw and additional K‐wiring: Results after 7.8 years – still an adequate procedure? BMC Musculoskelet Disord. 2017;18:339. 28778193 10.1186/s12891-017-1692-0 PMC5545010

[8] Lee YS , Lee S , Park JW , Kim CH , Kim J , Lee KH . Risk factors of loss of reduction after acromioclavicular joint dislocation treated with a hook plate. J Orthop Traumatol. 2023;24:43. 36961582 10.1186/s10195-023-00685-8 PMC10039153

[9] Kim DH , Ha KI , Park JH , Oh JH , Kim SH , Kim HJ . Comparison of TightRope system versus Bosworth screw for acute acromioclavicular joint dislocation. Arch Orthop Trauma Surg. 2016;136:203‐209.26602903

[10] Warth RJ , Martetschläger F , Gaskill TR , Millett PJ . Acromioclavicular joint separations. Curr Rev Musculoskelet Med. 2012;6:71‐78. 10.1007/s12178-012-9144-9 PMC3702768 23242975

[11] van Bergen CJA , van Bemmel AF , Alta TDW , van Noort A . New insights in the treatment of acromioclavicular separation. World J Orthop. 2017;8:861‐873. 29312844 10.5312/wjo.v8.i12.861 PMC5745428

[12] Guzman AJ , Rayos Del Sol S , Dela Rueda T , et al. Open acromioclavicular repair with a suture cerclage tensioning system: A case series. Cureus. 2023;15:e34018. 36811052 10.7759/cureus.34018 PMC9939279

[13] Struhl S , Wolfson TS , Kummer F . Axial‐plane biomechanical evaluation of 2 suspensory cortical button fixation constructs for acromioclavicular joint reconstruction. Orthop J Sports Med. 2016;4:2325967116674668. 28210644 10.1177/2325967116674668 PMC5298557

[14] Woodmass JM , Esposito JG , Thornton GM , Lo IKY . Complications following arthroscopic fixation of acromioclavicular separations: A systematic review of the literature. Orthop Surg. 2015;7:295‐302. 10.2147/OAJSM.S73211 PMC4401206 25914562

[15] Millett PJ , Warth RJ , Greenspoon JA , Horan MP . Arthroscopically assisted anatomic coracoclavicular ligament reconstruction technique using coracoclavicular fixation and soft‐tissue grafts. Arthrosc Tech. 2015;4:e583‐e587. 26900558 10.1016/j.eats.2015.06.007 PMC4722491

[16] Xue C , Zhang M , Zheng TS , et al. Clavicle and coracoid process drilling technique for truly anatomic coracoclavicular ligament reconstruction. Injury. 2013;44:1314‐1320. 23876623 10.1016/j.injury.2013.06.022

[17] Alkoheji M , Amis AA , El‐Daou H , Lee J . Acromioclavicular joint reconstruction implants have differing ability to restore horizontal and vertical plane stability. Knee Surg Sports Traumatol Arthrosc. 2021;29:3902‐3909. 34436636 10.1007/s00167-021-06700-x PMC8595167

